# A novel minimally invasive broken nail extractor for cannulated intramedullary nails: Trial and application in a few cases

**DOI:** 10.1097/MD.0000000000031549

**Published:** 2022-11-18

**Authors:** Zihong Zhou, Yan Jiang, Beichen Dai, Guoshu Mao, Yu Liu, Changbao Wei, Weidong Lin

**Affiliations:** a Department of Orthopaedics, Wuxi People’s Hospital Affiliated to Nanjing Medical University, Wuxi, Jiangsu, China; b Department of Radiology, Wuxi No. 9 People’s Hospital Affiliated to Soochow University, Wuxi, Jiangsu, China; c Department of Orthopaedics, Wuxi No. 9 People’s Hospital Affiliated to Soochow University, Wuxi, Jiangsu, China; d Department of Traumatology and Orthopaedics, Wuxi No. 2 Traditional Chinese Medicine Hospital, Wuxi, Jiangsu, China.

**Keywords:** broken intramedullary nail, broken nail extractor, hardware removal, minimally invasive

## Abstract

Successful and minimally invasive extraction of a broken distal end of an intramedullary nail is challenging. This study introduces a simple and reproducible technique for the extraction of broken cannulated intramedullary nails using a novel minimally invasive broken nail extractor. Five amputated adult lower-leg specimens were used to create models of the broken distal end of the cannulated intramedullary nails remaining in the medullary cavity of the distal tibia. Two orthopedic resident physicians with experience in tibial intramedullary nail implantation were selected to blindly extract the broken intramedullary nail using the novel minimally invasive broken nail extractor. The extraction outcome was assessed. The broken nail extractor was applied to 3 patients with broken intramedullary nails remaining in the medullary cavity of the distal tibia. In the lower-leg specimens, the extraction success rate was 100%, the median number of extraction times was 1.9 (range 1–3.5), and the median duration of extraction was 38 s (range 20–52 s). All the broken intramedullary nails in the 3 patients were successfully extracted without complications related to the surgery. The study shows that our technique is simple, reproducible, and has a high extraction success rate, but more case applications are needed to verify its effect.

## 1. Introduction

Intramedullary nailing of long bone fractures is an accepted technique with the advantages of not disturbing the fracture hematoma and biomechanical superiority over plating. However, the incidence of nonunion or delayed union of long bone fractures after intramedullary nailing is 10% to 18%.^[1–3]^ When nonunion or delayed union occurs, the intramedullary nail is subjected to considerable stress due to cyclic loading and the stress concentration at the fracture level and the locking hole, and fatigue failure of the nail can occur. Therefore, it is not uncommon for intramedullary nails to break in clinical practice. Broken nails remaining in the medullary cavity can be combined with bone nonunion or union. For postoperative nonunion of the fractured tibia and femur, closed replacement of the nail, including removal of the broken distal end of the nail, is becoming one of the preferred methods.^[4,5]^ For postoperative bone union, removal of internal implants, including broken implants, is also in line with the customs of some ethnic groups. Multiple techniques have been used to extract a broken distal end of the nail.^[5–12]^ Nevertheless, there is no guarantee that a particular extraction technique will be most effective for any given case. Therefore, it is incumbent on the surgeon to be familiar with a variety of intramedullary nail extraction techniques and have all potentially required equipment available. In this paper, we describe a simple reproducible extraction method for the broken distal end of cannulated nails using a novel minimally invasive broken nail extractor, which mainly reports trial results in cadaver specimens and their application in a few cases.

## 2. Materials and methods

### 2.1. Fabrication of the novel minimally invasive broken nail extractor

**Figure 1.**
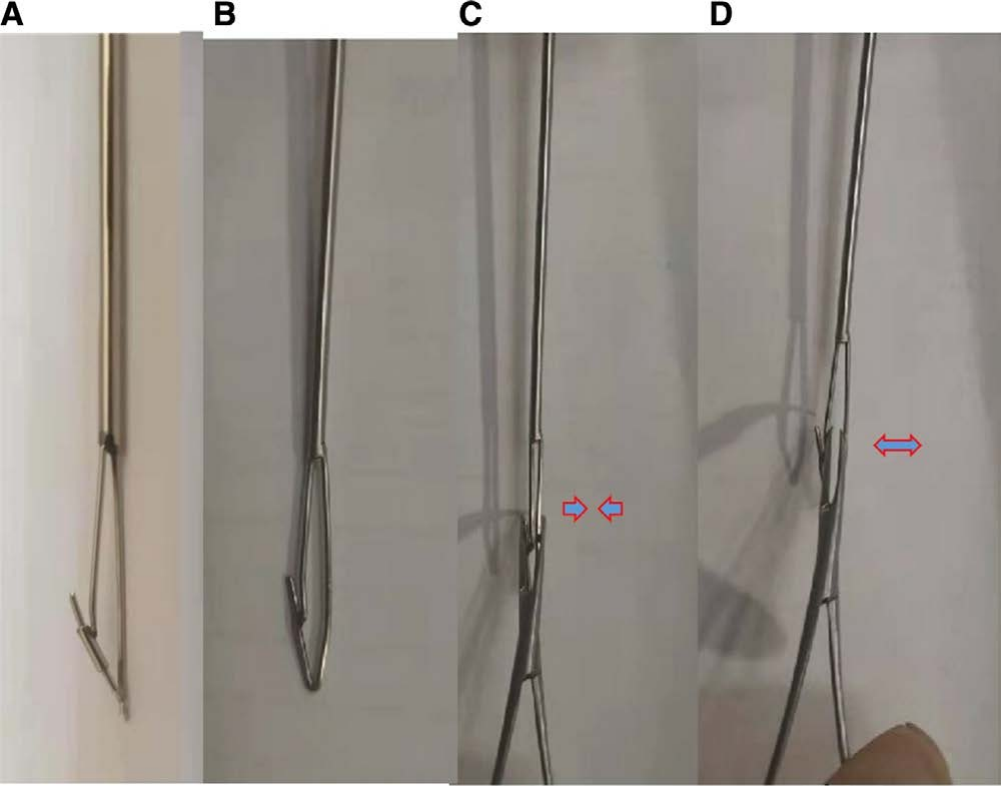
Novel minimally invasive broken nail extractor. (A, B) There were 2 types. (C, D) The diameter of the broken nail extractor at the barb can be adjusted.

The novel minimally invasive broken nail extractor for the broken distal end of cannulated intramedullary nails includes a guide wire and spear. The diameter of the guide wire is 2.8 mm. The spear consists of two Φ 1.0 mm Kirschner wires, with a barb with a diameter of 1 mm and a length of 2.5 mm on one side. The guide wire and spear were connected by welding. The diameter of the broken nail extractor at the barb can be adjusted according to the specific situation (Fig. 1).

### 2.2. Specimen and establishment of a tibial broken intramedullary nail model

The inclusion criteria were as follows: amputation at the distal thigh or knee joint level due to severe thigh trauma and (2) age >18 years old. Exclusion criteria: previous tibial surgery; complicated with fracture of the tibia; 1.9 m < height <1.5 m; and structural abnormality of tibia caused by congenital development or acquired disease.

**Figure 2.**
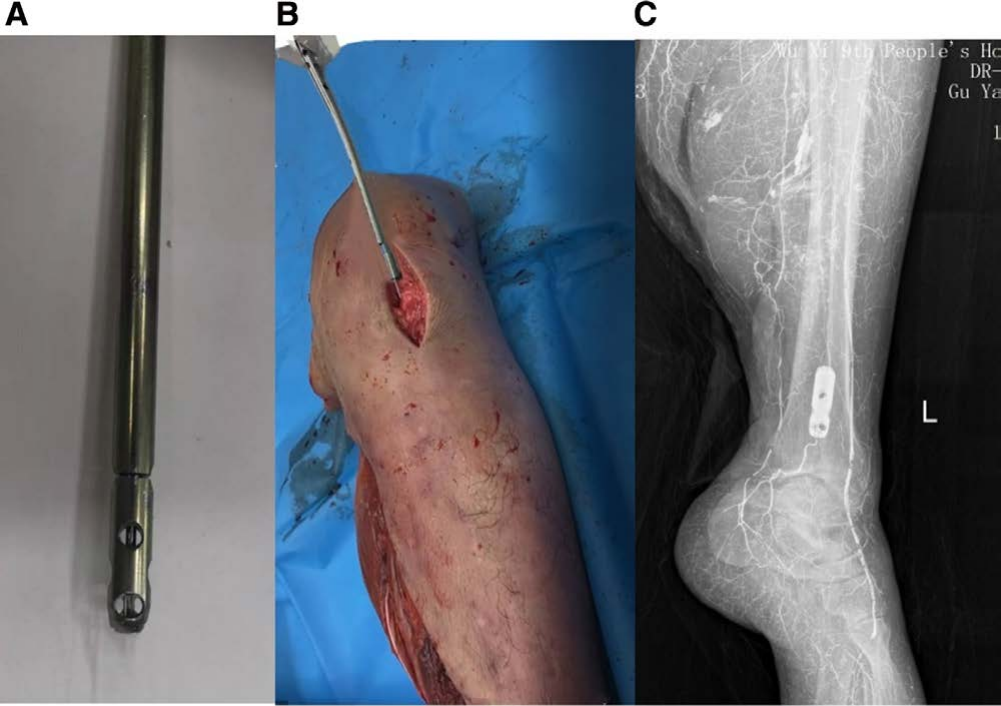
Establishment of the tibial broken nail model. (A) Breaking of the distal tibial intramedullary nail. (B) The broken front was placed in the medullary cavity of the distal tibia under the guidance of the guide pin. (C) X-ray showing the broken nail located in the medullary cavity of the distal tibia.

This study including trial and clinical application was approved by the ethics committee of our hospital. Informed consent was obtained from all subjects. All experiments were performed in accordance with the relevant guidelines and regulations. From January 1, 2019, to May 30, 2021, seven adult calf specimens were collected and cryopreserved, of which 5 met the inclusion criteria and were used. The age of the adults from which the specimens were obtained ranged from 20 to 58 years, with an average of 41 years. After thawing the specimens to room temperature, the lower leg was placed in a supine position on the OT table. A skin incision was made from the lower pole of the patella to the tibial tuberosity. A midline patellar tendon splitting approach was performed, and the entry portal was made on a soft spot with a curved awl, followed by guide wire insertion into the distal tibia. Sequential reaming of the canal with a Φ 11 mm drill bit along the guide wire. A Φ 10 mm tibial intramedullary nail (Jiangsu Aidier Medical Technology Co., Ltd., China) with a broken front was placed distal to the tibial medullary cavity along the guide wire. The guide wire and intramedullary nail were pulled out, and the distal end of the broken nail was left in the distal medullary cavity of the tibia, which was confirmed by X-ray images (Fig. 2).

### 2.3. Trial with the blind extraction method

Two orthopedic resident physicians with experience in tibial intramedullary nail implantation were selected to extract the broken nail using the novel broken nail extractor, which was inserted from the beginning of the proximal tibia to the distal tibia. When the physician felt that the broken nail was hooked, the broken nail extractor was pulled out through the canal toward where the nail entered the bone, which was defined as one time of extraction. If one time of extraction failed, the extraction procedure was repeated until the broken nail was removed; if the broken nail was not removed after 5 consecutive times, the procedure was defined as extraction failure. The extraction success rate, median extraction times and extraction duration were recorded. Each parameter was taken as the average value of two observers.

### 2.4. Clinical application

The novel minimally invasive broken nail extractor was applied to 3 patients with the distal end of the broken nail remaining in the distal tibial medullary cavity from January 1, 2020, to May 30, 2022. Among them, 2 cases had union, and 1 case had nonunion after internal fixation using an intramedullary nail. All patients were male, aged 32 to 58 years with a medium age of 48 years.

## 3. Results

**Figure 3.**
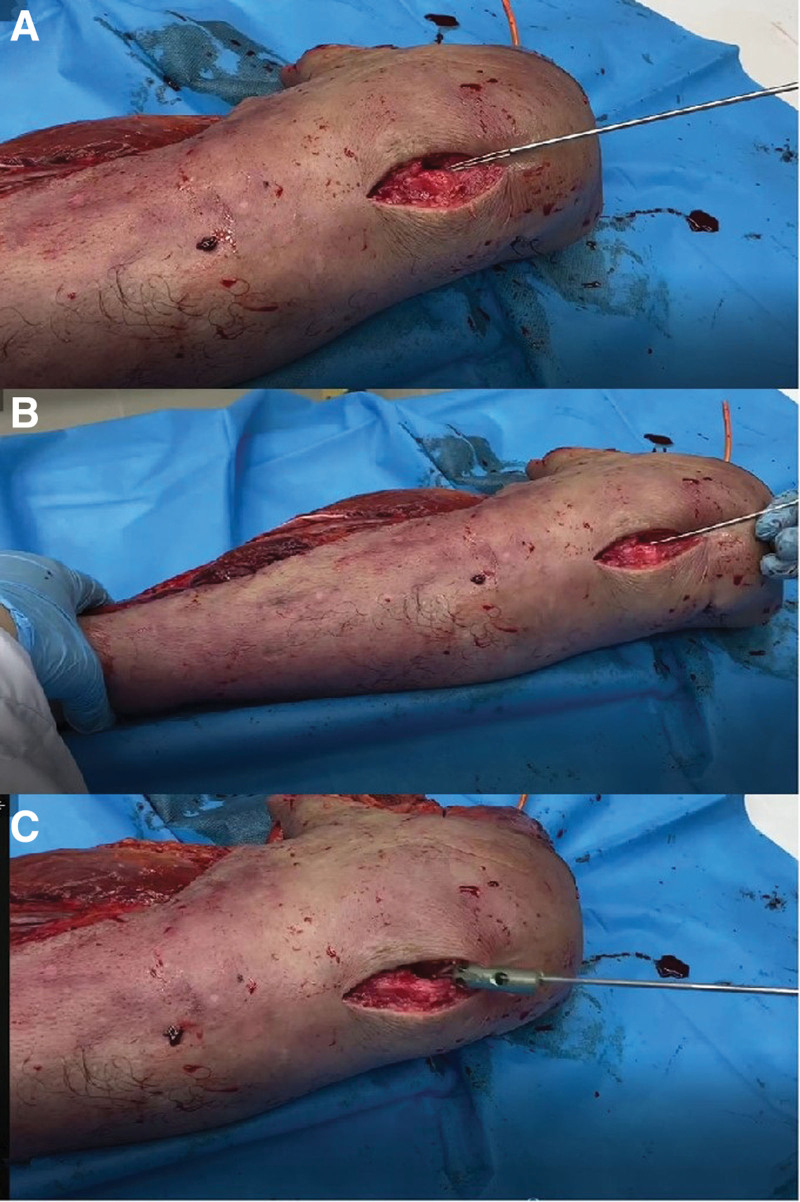
Trial of a novel minimally invasive broken nail extractor in a lower leg specimen with the blind method. (A) Inserting the broken nail extractor. (B) The broken nail extractor hooks the broken nail’s canal wall. (C) The broken nail extractor extracts the broken nail to the entry point.

In the specimen, the extraction success rate was 100%, the median extraction time was 1.9 (range 1–3.5), and the median extraction duration was 38 s (range 20–52 s). All broken cannulated intramedullary nails in the 3 cases were successfully extracted, the bone at the broken nail site was not fenestrated, and no adverse complications related to the surgery occurred (Fig. 3).

### 3.1. Typical case

**Figure 4.**
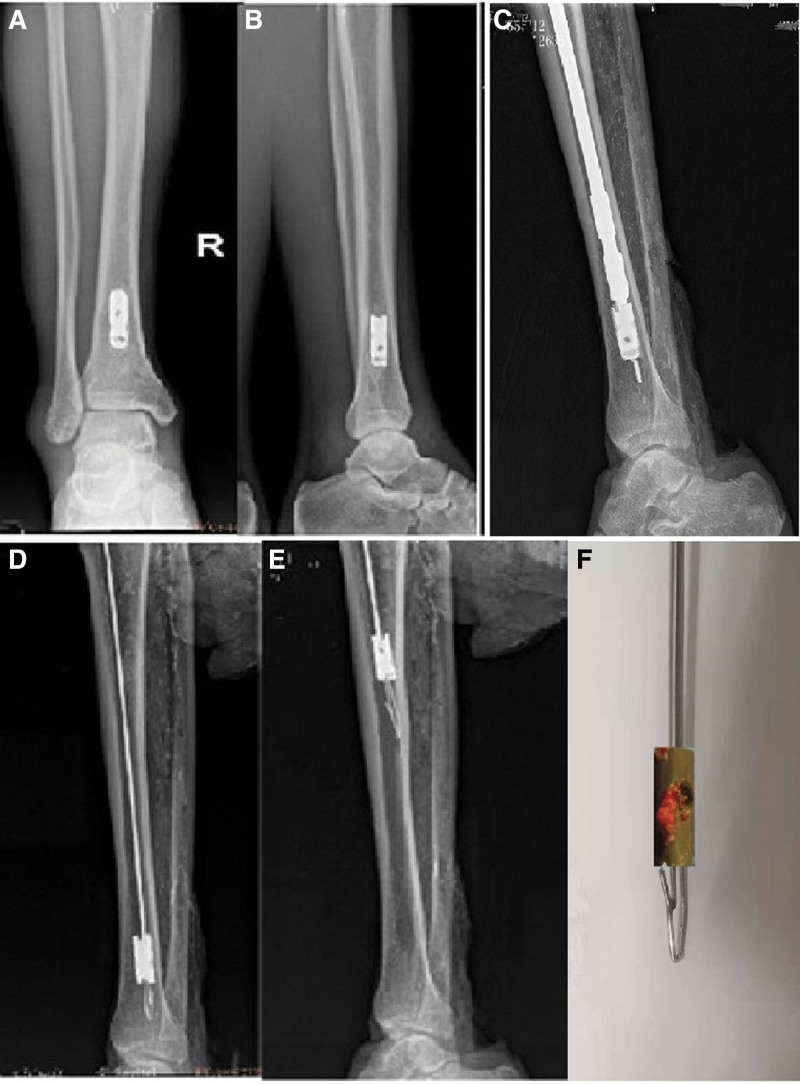
A 48-year-old male patient with fracture union and a distal broken segment of cannulated intramedullary nail. (A, B) X-ray showing fracture union and a distal broken end remaining at the tibial distal medullary cavity. (C) Intraoperative X-ray showing a drill reaming the medullary cavity along a guide needle. (D) Intraoperative X-ray showing that the broken nail extractor passed through the canal of the broken nail. (E) Intraoperative X-ray showing the barb of the broken nail hooking the canal wall. (F) The broken nail extractor extracted the broken nail.

A forty-eight-year-old male patient was admitted for the removal of all internal implants in his body. He had a closed tibial shaft fracture caused by a collision 15 years previously and was treated with closed reduction and intramedullary nail fixation. Delayed healing occurred after the surgery. The nail broke at the distal locking screw hole. The fracture healed after plaster fixation for 3 months. The internal implant was removed 3 years after the fracture, but the distal end of the broken intramedullary nail could not be removed using the hook extraction method and remained in the medullary cavity. Both surgeries were performed in another hospital. One year prior to admission, the patient underwent open reduction and internal fixation in our hospital due to a fracture of the right distal radius, which healed 2 months after the operation. His lower and upper limb function recovered well. It was known that the outer diameter of the broken nail was 10 mm, and the internal diameter was 3 mm from the medical documents of the hospital where the patient was treated. It can be seen from the preoperative X-ray film that the broken nail remaining in the intramedullary was hollow, and the internal diameter was larger than the isthmus of the medullary cavity (Fig. 4a and b). For the removal procedure, we made a hole at the entry point of the tibia for the intramedullary nail, inserted the guide wire, and reamed the tibial medullary cavity with a Φ 11 mm drill bit along the guide wire (Fig. 4c). The residue was flushed and aspirated after reaming. We adjusted the diameter of the barb on the spear of the novel minimally invasive broken nail extractor to 3 mm, inserted the spear into the medullary cavity and passed it through the canal of the broken nail (Fig. 4d). When the extractor was pulled back, the barb hooked the canal wall, (Fig. 4e), and the broken nail was successfully extracted (Fig. 4f).

## 4. Discussion

Different techniques and methods have been described for extraction of the distal end of a broken cannulated intramedullary nail.^[6–12]^ According to whether the bone at the broken nail site is fenestrated, these techniques can be divided into two categories: minimally invasive and fenestration extraction methods.

The fenestration extraction method has a high success rate and basically avoids radiation exposure, but the disadvantage is that it creates relatively large area of surgical trauma.

The interference fit guide-wire method and hook removal methods are the main invasive extraction methods.^[6,7,12,13]^ The interference fit-guide wire method entails passing a ball-tipped guide wire and a nontipped guide wire or Kirschner wire through the nail canal, filling the canal as much as possible and extracting the broken nail using the resistance generated by the friction between the enlarged ball at the front of the wire and the canal wall.^[6,7]^ The disadvantage of this method is that it is very difficult for the nontipped guide wire and the Kirschner wire to pass through the canal of the broken nail along with the ball-tipped wire. The hook extraction method involves hooking a guide wire through the broken nail canal and then extracting the broken nail. The disadvantage of the hook method is that it is very difficult for the hooked guide wire to pass through the broken nail canal. The typical case in the study was a patient that experienced failed hook extraction. Minimally invasive extraction methods require multiple X-ray fluoroscopies, which subject operators and patients to large doses of X-ray radiation, and their extraction success rate is not high.

Our technique is simple to manufacture, and only one guide pin and one Kirschner wire must be welded. Its design is not only reasonable, but the success rate of extraction is also high. The tip of the extractor is relatively small and is located in the center of the front of the extractor so that it can easily enter the canal of the broken nail and guide the wide spear into the canal. The diameter of the spear at the barb could be reduced and expanded. After compression, the reduced diameter was ≤3 mm, allowing the body to pass smoothly through the canal of the broken nail. After passing through the canal, the body diameter increased to >3 mm, and the barb could easily hook the canal wall of the broken nail. The width of the barb is increased by nearly 2.0 mm after full expansion of the barb, not exceeding the thickness of the wall of the broken nail, so the barb does not hook the medullary cavity wall and get stuck. The connection between the barb and Kirschner wire provides appropriate resistance or supporting force to allow the broken nail to be extracted.

The key points of operation are as follows: when it is expected that the broken nail extractor will encounter great resistance when passing through the medullary cavity, especially at the isthmus, a drill must be used to ream the medullary cavity. Second, the diameter of the broken nail extractor at the barb should be adjusted according to the internal diameter of the broken nail to be consistent with or slightly larger than the internal diameter of the broken nail. Otherwise, if the diameter is too large, it is not easy to enter the canal of the broken nail tube; if the diameter is too small, it is not easy to hook the tube wall.

## 5. Limitations of the study

There was no comparison with other methods; only a few cases were used in this study. Therefore, comparisons with other methods and more case applications are needed to verify its effect.

## 6. Conclusions

Our technique is a simple reproducible alternative that has a high extraction success rate, but more case applications are needed to verify its effect.

## Author contributions

**Conceptualization:** Zihong Zhou, Yan Jiang, Changbao Wei.

**Data curation:** Beichen Dai, Guoshu Mao.

**Methodology:** Yu Liu.

**Resources:** Zihong Zhou, Guoshu Mao.

**Supervision:** Yan Jiang.

**Writing – review & editing:** Chnagbao Wei.

Changbao Wei: 0000-0002-1381-0710
